# Comprehensive phenomics and vegetative yield analysis of global kale (*Brassica oleracea* var. *acephala*) germplasm in controlled environment agriculture

**DOI:** 10.1186/s12870-026-08380-6

**Published:** 2026-02-17

**Authors:** Adrian Ming Jern Lee, Ting Xiang Neik, Shuang Song, Kwai Wei Chan, Seam Choon Law, Pei-Wen Ong, Ethan Tze Cherng Lim, Fook Tim Chew

**Affiliations:** 1 Department of Biological Sciences, National University of Singapore, 14 Science Drive 4, Singapore, 117543 Singapore; 2 Research Centre On Sustainable Urban Farming (SUrF), National University of Singapore, 14 Science Drive 4, Singapore, 117543 Singapore; 3 Lee Hiok Kwee Functional Genomics Laboratories, Block S2, Level 5, 14 Science Drive 4, Lower Kent Ridge Road, Singapore, 117543 Singapore

**Keywords:** *Brassica oleracea* var*. acephala*, Indoor farming, Phenomics, High-throughput phenotyping, Plant breeding

## Abstract

**Background:**

Kale (*Brassica oleracea* var. *acephala*) is a high value leafy vegetable with an extensive domestication history and germplasm diversity, making it an ideal target for genetic improvement. To meet growing food security needs particularly with controlled environment agriculture (CEA) systems, specialized breeding strategies are required. The goal of this study was to survey the phenotypic architecture of a global kale germplasm collection under commercial CEA conditions. This study establishes a phenotypic baseline and serves as a hypothesis generating resource for future genetic and physiological studies in kale and other leafy vegetables grown under CEA.

**Results:**

A total of 203 kale accessions were phenotyped for 113 quantitative traits using high-throughput phenotyping methods. Significant differentiation was observed across all traits, with coefficient of variation ranging from 2.5% to 180.7%, confirming broad genetic variability among accessions. Trait correlation networks and hierarchical clustering grouped phenotypes into seven biologically corresponding modules including leaf, stem and root morphology, plant architecture, hyperspectral indices, and seedling growth. These modules highlight coordinated phenotypic patterns among traits. Integrative yield analyses combining partial least squares variable importance in projection with differential trait analysis identified 28 phenotypes most strongly associated with total aboveground fresh weight, a robust proxy for CEA vegetative yield. Principal component analysis further distilled these traits into three orthogonal components explaining 87.1% of total yield variation. These components represented modules related to plant organ size, canopy structure, and density, emphasizing their biological contribution to harvestable biomass.

**Conclusions:**

This study generates a foundational phenomics resource and comprehensive dissection of kale’s yield architecture under CEA conditions. The composition of traits identified constitutes a targeted set of breeding traits to be further validated for improved leafy vegetable yield. By integrating large-scale germplasm resources with phenomics, this work establishes the utility of a high-throughput phenotypic analysis for further leafy crop research and improvement.

**Supplementary Information:**

The online version contains supplementary material available at 10.1186/s12870-026-08380-6.

## Background

Global food security faces mounting challenges from supply chain disruptions, climate change, urbanization, and population growth [1–5]. Between 2010 and 2050, food demand is projected to increase by 35–56% while arable land availability continues to decline, particularly in densely populated cities [5]. In Singapore, agricultural land has halved in the past two decades, now accounting for less than 1% of the nation state’s total land use [5, 6].

Controlled Environment Agriculture (CEA), encompassing vertical and indoor farming, is a promising solution for enhancing urban food production [6, 7]. These systems offer key advantages such as efficient land use and reliable, year-round local supply. However, their broader adoption remains limited due to high operational costs. Electricity and other input resources particularly affect farm profitability [8, 9]. While innovative engineering approaches have been developed to mitigate some of these costs, relatively little attention has been directed toward improving vegetable cultivars specifically adapted to CEA conditions [10]. Enhancing yield potential, light-use efficiency, and crop quality has been identified as some of the most effective strategies to improve the economic viability and sustainability of CEA systems [7, 8, 10, 11].

Among leafy vegetables, kale (*Brassica oleracea* var. *acephala*) is widely cultivated in CEA systems due to its fast growth cycle, high adaptability to vertical farming systems and strong consumer demand [1, 7, 12, 13]. Often marketed as a “superfood,” kale is one of the most nutrient dense leafy greens [14, 15]. However, most cultivars currently grown in CEA originate from open field breeding programs. While suitable for indoor cultivation, these varieties are not yet optimized for CEA [10, 16]. Developing specialized cultivars with traits such as compact architecture, efficient nutrient uptake, adaptability to artificial lighting, and enhanced biomass production could substantially improve CEA productivity and profitability [8, 10, 11, 16, 17]. Furthermore, sensory traits such as flavor, leaf texture, and coloration offer opportunities for novel kale varieties that capture greater consumer acceptance and market competitiveness [8, 16, 18].

Breeding for CEA specific cultivars has been largely constrained by challenges in phenotyping. Conventional approaches are labor intensive and limited in scalability [19, 20]. The advent of high-throughput phenotyping (HTP) now enables multi-trait, high resolution evaluation of diverse plant germplasm, providing a powerful avenue to dissect phenotypic variation and accelerate breeding efforts [19, 20]. HTP has been recommended as a means to harness the genetic diversity of diverse germplasms that are otherwise hard to screen [21]. For example, soybean breeding plans were enhanced using HTP imaging to more effectively select superior genotypes [22]. In tomato germplasm, fruit diversity was studied using HTP methods to better enrich pre-breeding programs [23].

In this study, we applied a comprehensive HTP approach to a globally diverse panel of 203 kale accessions. Our analysis encompassed 113 quantitative traits measured under controlled environment conditions, generating a foundational phenomics resource under a hypothesis generating framework. The objectives of this study were thus to: (i) quantify phenotypic variation and heritability across global kale germplasm under CEA conditions, (ii) identify modular organization of phenotypic traits, and (iii) highlight candidate phenotypic traits relevant for yield improvement and CEA specific breeding. By integrating a broad global germplasm with large-scale phenomics, we provide new insights into the trait architecture of kale and identify candidate phenotypic traits of interest for future breeding research.

## Materials and methods

### Germplasm collection

A total of 203 *B. oleracea* var. *acephala* accessions were collected from international gene banks and commercial sources (Table 1). Detailed metadata of each accession, including country of origin, cultivar, and source, are provided in Supplementary Table 1. Seeds were maintained at 20 °C and 15% relative humidity (RH).

**Table 1:** Geographic distribution of 203 *B. oleracea* var. *acephala* accessions included in the study

Continent	Gene bank	Commercial
Africa	0	1
Asia	4	11
Europe	104	7
North America	7	20
Oceania	2	0
Unknown	1	46
Total	118	85

### Experimental design and growth conditions

Plants were cultivated under CEA conditions using an incomplete randomized block design, following previously established screening protocols [24]. Twelve experimental blocks were conducted, each comprising about 18 accessions with a minimum of three replicates per accession. All accessions underwent prior seed germination testing to ensure successful establishment, allowing at least three replicates of all 203 accessions to be phenotyped. To replicate CEA farm conditions, plants were grown on the SANANBIO Radix vertical hydroponic module (San’an Optoelectronics Co., Ltd., Xiamen, China). Environmental conditions of the growth room were maintained at 21 ± 1 °C, 70–80% RH, and a 16-h light/8-h dark photoperiod protocol. Hydroponic nutrient solution conductivity was adjusted across developmental stages, from 1.0 S/m (seedling), 1.5 S/m (hardening) to 2.0 S/m (maturing).

### High-throughput phenotyping

Phenotyping was conducted at 14 (seedling), 21 (hardening), and 28 (maturing) days after sowing (DAS) on at least three randomly selected plants per accession. This process involved both non-destructive and destructive measurements. Phenotyping was performed using five complementary approaches: Planteye 3D scanning (42 traits), EasyPCC image analysis (8 traits), ImageJ manual image measurements (28 traits), derivative based metrics (30 traits), and direct manual measurements (8 traits), yielding a total of 113 quantitative traits (Supplementary Table 2). Non-destructive imaging was performed using the Planteye F500 multispectral 3D laser scanner (Phenospex, Heerlen, Netherlands) on all three phenotyping days. Top-down canopy photographs were acquired at 21 DAS with a 12-megapixel (MP) smartphone camera and analyzed using the EasyPCC software [25]. Destructive phenotyping was performed at 28 DAS when each plant was harvested, and total aboveground fresh weight was recorded as the primary yield proxy. The largest leaf of each plant was imaged with the same 12 MP smartphone camera and analyzed using ImageJ v1.53 [26], stem and root images were taken and measured using the same method. Stem and root dry weights were subsequently obtained following oven drying at 60 °C for three days. Derivative traits (including ratios, indices, and composite measures) were calculated from these direct measurements. All phenotyping on 14, 21, and 28 DAS was performed on the same individual plants to enable longitudinal tracking of trait development.

### DNA extraction and heritability analysis

Tissue samples from the leaves of every plant were collected and genomic DNA was extracted using the DNAeasy® Plant Mini Kit (Qiagen). They underwent whole genome resequencing using the NovaSeq 6000 Sequencing System at 7 × sequencing depth. Paired-end reads were aligned to the published *Brassica oleracea* TO1000 reference genome [27] using Burrow-Wheeler Aligner version 0.7.13 ‘MEM’ algorithm [28]. Alignment files were sorted using SAMtools 1.3 [29] and variants called using the ‘mpileup’ function with BASE and MAP quality options (20 and 30 respectively). Single nucleotide polymorphism (SNP) variants were filtered using PLINK version 1.0 [30] according to the following criteria to obtain the final genotyping data set: Only SNPs, > 0.05 minor allele frequency (MAF) and < 10% missingness rate. Narrow-sense SNP based heritability (h^2^) was estimated for all traits using the restricted maximum likelihood (REML) framework implemented in GCTA [31]. GCTA operates on the genomic relationship matrix (GRM), modelling block-level random effects from the incomplete block design is thus not directly applicable within this framework. To ensure appropriate model specification, population structure was controlled for by including the first ten genomic principal components as fixed covariates in the REML model. Standard errors of all h^2^ estimates were suitably recorded. Whole genome resequencing data were generated for use in heritability estimation and as a genomic resource for future analyses; only SNP-based h^2^ estimation is presented here.

### Statistical analysis

All statistical analyses were performed in RStudio [32] and Microsoft Excel (Version 16.102.1). Descriptive statistics were calculated from raw data. Missing data were imputed using the missForest package [33]. Ordered quantile normalization was applied to trait distributions using the bestNormalize package [34] to reduce scale effects, skewness, and outlier influence. Missing data imputation and trait normalization was performed to stabilize correlation and dimension reduction analyses rather than to infer individual level trait effects. Imputed values were not interpreted directly, and trait relationships involving highly imputed variables were interpreted conservatively. Such data pre-processing and normalization is greatly important for accurate downstream analysis [35, 36].

Significant trait variation across accessions was statistically confirmed using the Kruskal–Wallis test (*p <* 0.05) on raw accession means. Trait relationships were evaluated using Pearson’s correlation coefficient, and relationships with r > ± 0.6 were used to construct correlation networks via the igraph package [37]. The conservative correlation threshold was applied to avoid dense, uninterpretable networks dominated by weak correlations. Given that data were imputed and normalized prior to analysis, conservative correlation thresholds prevented visualization of spurious correlations. The Louvain community detection algorithm was applied to partition the network into biologically relevant trait modules [38]. While formal robustness analysis was not performed, major trait groupings were qualitatively conserved across thresholds and reflected coherent biological trait categories. These modules were further subdivided by hierarchical clustering with the pheatmap function [39]. These algorithms have been shown to be useful in separating large scale biological networks [40] and should be further explored [41]. However, because module detection largely depends on the chosen similarity metric and clustering algorithm, alternative approaches could yield slightly differing module boundaries. The trait modules identified here should thus be interpreted as context dependent organizational frameworks that summarize patterns of trait covariance, rather than definitive or mechanistically resolved biological units. Mean r^2^ (Coefficient of Determination) within modules and submodules were calculated by averaging all pairwise trait r^2^ within each module.

Yield associated traits were robustly identified by combining two complementary statistical approaches. Firstly, partial least squares (PLS) regression, implemented with the pls package [42] (the optimal number of components was selected based on tenfold cross-validation of the plsr function) where variable importance in projection (VIP) scores (> 1) were used to rank predictors of total fresh weight (TFW). Secondly, differential trait analysis comparing the top and bottom 10th percentile of accessions for TFW, conducted with the limma package [43]. Block effects were not explicitly modeled due to the incomplete block design and lack of block-level replication. Instead, normalized values helped mitigate potential batch or experimental confounding. Significant traits were defined at false discovery rate (FDR) < 0.05 and log₂FC > 1. Results were visualized using the EnhancedVolcano package [44]. Traits consistently identified by both methods were designated as key yield contributing traits. Principal Component Analysis (PCA) was applied to this reduced set of traits to extract orthogonal components, which served as candidate phenotypic traits of interest for vegetative yield improvement in kale. These analyses were conducted under a limited CEA condition and time points. They were intended to identify traits statistically associated with yield in this context. They are not intended as predictive models, and no external validation or cross-environment testing was performed.

## Results

### Phenotypic variation and trait heritability

Significant phenotypic variation was observed across accessions for all traits (Kruskal–Wallis, *p <* 0.05). Coefficient of variation (CV) spanned a wide range, from highly conserved traits such as Blade Solidity (BSOL; 2.5%) to highly plastic traits such as Digital Biomass at 14 DAS (DBM14; 180.7%) (Supplementary Table 3). Thirty-six traits exhibited CV values above 50% and are summarized in Table 2. These highly variable traits were distributed across all phenotyping platforms. Spectral indices including Normalized Pigment Chlorophyll Ratio Index (NPCI) and Plant Senescence Reflectance Index (PSRI), explaining differences in plant pigmentation, also displayed high variability. Full descriptive statistics for all 113 phenotypes are provided in Supplementary Table 3.

**Table 2:** Descriptive statistics and heritability calculations for 36 quantitative traits exhibiting CV > 50%, in order of decreasing CV

Trait	Abbreviation	Mean^a^	SD^b^	Min	Max	CV^c^	Heritability
DAS 14 Digital Biomass^d^	DBM14 (cm^3^)	13.47*	24.34	0.00	343.01	180.68	0.59
Leafing Intensity	LINT (g^−1^)	312.92*	538.22	20.27	11,000.00	172.00	0.45
DAS 14 Leaf Area^d^	LA14 (cm^2^)	10.71*	16.28	0.00	155.33	151.97	0.53
DAS 14 Leaf Area Projected^d^	LAP14 (cm^2^)	8.52*	12.78	0.00	117.54	150.00	0.56
DAS 14 Leaf Area Index^d^	LAI14 (No unit)	0.01*	0.02	0.00	0.17	145.01	0.52
Specific Root Length	SRL (cm/g)	173.22*	229.28	10.70	3678.17	132.37	0.55
Root Length-Leaf Area Ratio	RLLA (cm^−1^)	0.78*	1.02	0.07	22.29	129.77	0.50
DAS 21 Digital Biomass^d^	DBM21 (cm^3^)	174.91*	197.37	0.00	1615.58	112.84	0.60
DAS 21 Plant Senescence Reflectance Index^d^	PSRI21 (No unit)	0.03*	0.03	−0.21	0.31	105.06	0.42
DAS 21 Leaf Area Projected^d^	LAP21 (cm^2^)	32.18*	28.68	0.00	186.99	89.11	0.47
DAS 21 Leaf Area Index^d^	LAI21 (No unit)	0.05*	0.04	0.00	0.28	83.74	0.47
DAS 21 Leaf Area^d^	LA21 (cm^2^)	48.11*	40.21	0.00	251.27	83.59	0.50
Total Fresh Weight	TFW (g)	23.14*	19.19	0.54	139.78	82.93	0.95
DAS 28 Plant Senescence Reflectance Index^d^	PSRI28 (No unit)	0.02*	0.01	−0.11	0.15	76.73	0.31
Stem Fresh Weight	SFW (g)	0.63*	0.48	0.00	5.84	76.68	0.59
Root-Stem Ratio	RST (No unit)	6.39*	4.88	0.24	106.86	76.35	0.30
Specific Root Area	SRA (cm^2^/g)	180.62*	136.65	17.13	4493.44	75.66	0.21
Surface	SF (Pixels)	819,661*	584,541	22,519	3899,122	71.31	0.99
DAS 21 Normalised Pigment Chlorophyll Ratio Index^d^	NPCI21 (No unit)	0.09*	0.06	−0.10	0.44	69.16	0.51
Stem Dry Weight	SDW (g)	0.06*	0.04	0.00	0.44	66.98	0.63
Coverage	CV (No unit)	0.08*	0.05	0.00	0.32	65.46	0.99
DAS 14 Plant Senescence Reflectance Index^d^	PSRI14 (No unit)	0.07*	0.04	−0.20	0.27	59.94	0.57
Root Dry Weight	RDW (g)	0.34*	0.20	0.01	2.27	59.67	0.76
Blade Area	BA (cm)	57.01*	32.09	1.02	284.23	56.30	0.69
Specific Leaf Area	SLA (cm^2^)	60.26*	33.09	1.05	290.75	54.91	0.72
Root Perimeter-Area Ratio	RPAR (cm^−1^)	2.61*	1.43	0.57	13.02	54.83	0.37
Stem Area	STA (cm^2^)	1.90*	1.03	0.11	10.89	54.33	0.35
Blade Convex Hull Area	BCHA (cm^2^)	63.46*	34.31	1.16	303.45	54.07	0.75
Stem Convex Hull Area	STCHA (cm^2^)	2.37*	1.28	0.14	15.74	53.93	0.46
DAS 28 Normalised Pigment Chlorophyll Ratio Index^d^	NPCI28 (No unit)	0.06*	0.03	−0.07	0.41	52.15	0.43
Specific Leaf Convex Hull Area	SLCHA (cm^2^)	74.91*	39.01	1.28	332.40	52.08	0.66
Root Area	RA (cm^2^)	44.34*	23.03	3.47	282.54	51.95	0.46
Root Width-Length Ratio	RWL (No unit)	0.13*	0.07	0.01	0.91	50.93	0.25
DAS 14 Light Penetration Depth^d^	LPD14 (cm)	0.71*	0.36	0.00	2.88	50.75	0.69
DAS 21 Light Penetration Depth^d^	LPD21 (cm)	2.08*	1.05	0.00	6.52	50.35	0.43

^a^*Indicates significant differences among accession means at *p <* 0.05 in the Kruskal–Wallis test

^b^*SD* Standard deviation

^c^*CV* Coefficient of variation

^d^*DAS* Days after sowing

Several traits exhibited comparatively low variability, including leaf orientation (Leaf Angle and Inclination across timepoints, CV < 16%) and spectral indices such as Normalized Difference Vegetation Index (NDVI28; 5.6%) and hue at DAS28 (HUE28; 7.9%). Leaf shape descriptors such as Blade Ellipticalness Index (BEI) and Blade Solidity (BSOL) exhibited CV values below 12%, indicating relatively conserved shape features compared to organ size and biomass traits.

h^2^ estimates ranged widely across traits (0.14 to 0.95) (Table 2, Supplementary Table 3). Traits related to organ size and plant architecture, such as TFW, Specific Leaf Width, Root Dry Weight, Blade Convex Hull Area, Specific Leaf Area, Blade Convex Hull Perimeter, DAS 28 Leaf Inclination, DAS 14 Light Penetration Depth and Solidity exhibited moderate to high heritability (h^2^ = 0.69–0.95). These traits therefore show strong genetic control within this CEA environment. In contrast, many spectral indices and derivative descriptors displayed lower heritability (h^2^ < 0.3), suggesting stronger developmental or microenvironmental influence. The observed distribution of CV and h^2^ thus highlights which trait classes provide the strongest basis for genetic selection for CEA. No genome-wide association studies (GWAS), genomic prediction, or population genetic analyses were conducted in this study.

### Trait correlation and module identification

**Fig. 1:**
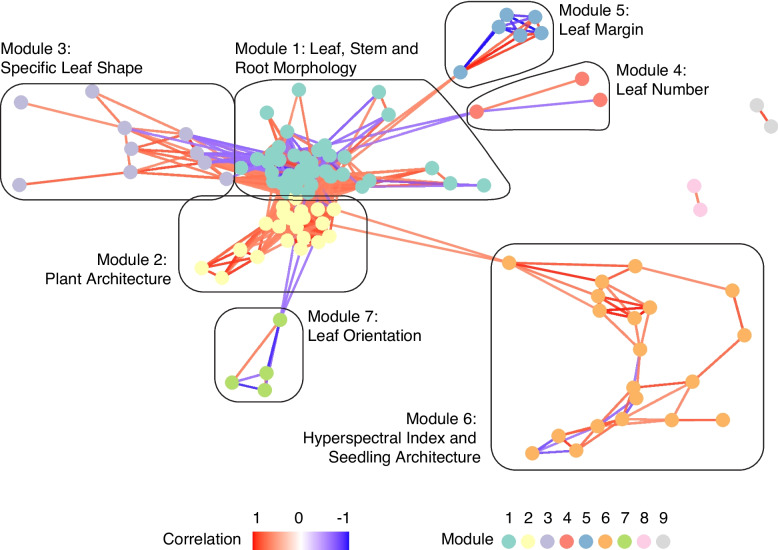
Kale trait correlation network identifying seven modules via Louvain community detection. Modules 8 and 9, each containing only two traits, were excluded from further analysis. Module 1: leaf, stem, and root morphology (*n =* 39), 2: plant architecture (*n =* 21), 3: leaf shape (*n =* 10), 4: leaf number (*n =* 3), 5: leaf margin (*n =* 6), 6: hyperspectral indices and seedling architecture (*n =* 20), 7: leaf orientation (*n =* 4)

Correlation network analysis clustered 107 of the 113 quantitative traits, revealing nine preliminary modules (Fig. 1). Six traits (Aspect Ratio, DAS 14 Leaf Angle, DAS 14 Leaf Inclination, Root to Stem Weight Ratio, RS Ratio and Stem Water Content) did not display any strong relationships with one another and were hence excluded. Modules 8 and 9, each containing only two traits, were excluded from further analyses due to limited interpretability. The seven remaining modules analyzed were categorized as: (1) leaf, stem, and root morphology (*n =* 39); (2) plant architecture (*n =* 21); (3) leaf shape (*n =* 10); (4) leaf number (*n =* 3); (5) leaf margin (*n =* 6); (6) hyperspectral indices and seedling architecture (*n =* 20); and (7) leaf orientation (*n =* 4) (Table 3) for biological interpretation. Together, they encompassed a total of 103 traits.

**Table 3:** Summary of trait modules, biological significance and mean correlations

Module	Module name	Biological Significance	Number of traits	Mean r^2^	Pairwise submodule correlation (r)
1	Leaf, Stem and Root Morphology	-	39	0.52	-
1.1	Organ Shape	Leaf, stem and root outline and form	9	0.40	−0.60
1.2	Organ Size	Quantitative dimensions of the leaf, stem and root	30	0.47	
2	Plant Architecture	-	21	0.43	-
2.1	Plant Canopy	Ground coverage of entire plant structure	5	0.78	0.58
2.2	Plant Density	Density and leaf area of entire plant structure	16	0.65	
3	Specific Leaf Shape	Features representing the overall shape of the leaf	10	0.34	-
4	Leaf Number	Number of leaves and its derivative traits	3	0.35	-
5	Leaf Margin	Quantitative representation of the lobation of the leaf	6	0.80	-
6	Hyperspectral Index and Seedling Architecture	-	20	0.21	-
6.1	Hyperspectral Index	Spectral indexes representing colour and health of plants	10	0.32	−0.15
6.2	Seedling Architecture	Plant architecture traits at seedling stage	10	0.35	
7	Leaf Orientation	Angle and erectness of plant’s leaves	4	0.57	-

**Fig. 2:**
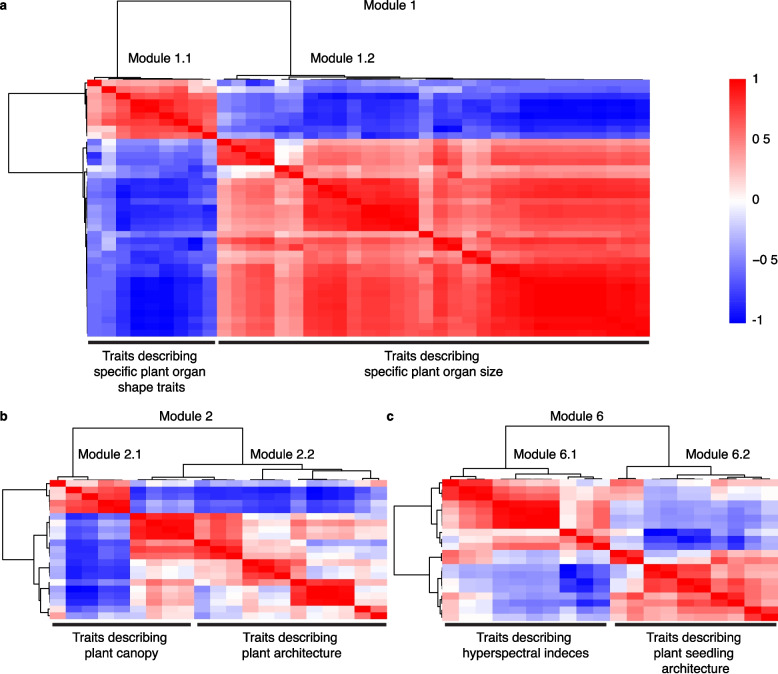
Representative trait module heatmaps identified. **a** Module 1: leaf, stem, and root morphology; **b** Module 2: plant architecture; and **c** Module 6: hyperspectral indices and seedling architecture of internal trait-trait correlations. Submodules are indicated. Sub-module 1.1: Organ Shape, 1.2: Organ Size, 2.1: Plant Canopy, 2.2: Plant Architecture, 6.1: Hyperspectral Index, 6.2: Seedling Architecture. All remaining heatmaps can be found in Supplementary Fig. 1

Module-specific heatmaps revealed additional hidden structure within several major modules: Modules 1, 2 and 6 (Fig. 2). Module 1 (leaf, stem, and root morphology) separated into two sub-modules: 1.1 (organ shape) (*n =* 9) and 1.2 (organ size) (*n =* 30) (Fig. 2a, Table 3). Sub-module 1.1 included traits such as Blade Eccentricity, Solidity, and shape ratios with moderate internal coherence (r^2^ = 0.40). These traits reflect geometric variations on organ shape. Sub-module 1.2 comprised size and biomass related traits including Leaf Area, Stem Fresh weight, and Root Dry Weight. Modules 1.1 and 1.2 were negatively correlated with one another (*r =* −0.60), an indication of the inverse relationship between organ shape and size. Module 2 (plant architecture) separated into sub-modules 2.1 (canopy related traits) (*n =* 5) and 2.2 (density related traits) (*n =* 16) (Fig. 2b, Table 3). Traits in sub-module 2.1, such as Leaf Area (Projected), Leaf Area Index, and Canopy Coverage, showed overall strong coherence with one another (r^2^ = 0.78) and reflect the spatial occupation and light interception potential of the plant canopy. Sub-module 2.2 included density related traits describing leaf overlap and compactness (r^2^ = 0.65) (Fig. 2b). The two submodules were positively correlated (*r =* 0.58), indicating their possible contribution to one another. Module 6 (hyperspectral indices and seedling architecture) was subdivided into 6.1 (spectral indices across developmental stages) (*n =* 10) and 6.2 (seedling architectural traits) (*n =* 10) (Fig. 2c, Table 3). Modules 3–5 and 7 (leaf shape, leaf number, leaf margin and leaf orientation) (Supplementary Fig. 1) primarily contained specialized morphological traits (Table 3). These traits were largely relevant to leaf specific traits. Representative traits for each module and sub-module, along with their biological interpretations, are summarized in Table 3.

### Determinants of vegetative yield

**Fig. 3:**
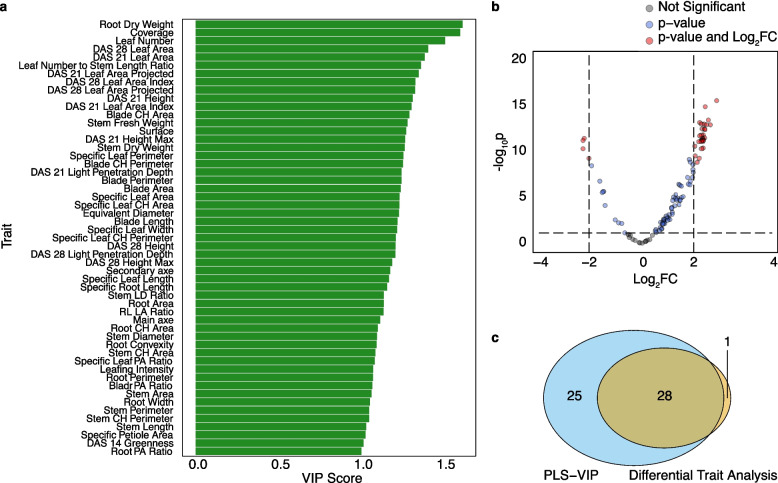
Identification of yield associated traits in kale. **a** PLS-VIP scores ranking phenotypes according to their contribution to yield determination. **b** Volcano plot from differential trait analysis comparing high against low yielding accessions, highlighting significantly enriched traits (red). **c** Venn diagram showing overlap of 28 common traits identified by PLS-VIP and differential analysis

To identify the traits most strongly associated with vegetative yield (measured as TFW), we applied two complementary approaches: PLS-VIP and differential trait analysis between high and low yielding accessions (top and bottom 10th percentile) (Fig. 3).

The PLS-VIP analysis ranked traits based on their contribution to explaining variation of the TFW trait. Forty-three phenotypes were identified with VIP scores above the threshold of 1 (Fig. 3a). These traits included major morphological measures such as Leaf Area, Blade Perimeter, and Height, as well as canopy traits (Coverage, Leaf Area (Projected), Light Penetration Depth) and biomass measures (Stem Fresh Weight, Root Dry Weight). Differential trait analysis was performed by comparing accessions in the top and bottom yield quartiles and identified twenty-nine phenotypes (FDR < 0.05, log₂FC > 2) (Fig. 3b). Integrating both approaches yielded a consensus set of twenty-eight phenotypes consistently associated with yield (Fig. 3c and Table 4). These traits collectively represent robust determinants of vegetative biomass, capturing morphological, architectural, and physiological aspects of plant growth.

**Table 4:** Loadings of 28 consensus yield associated traits on PC1-PC3, collectively explaining 87.1% of the variation in TFW

Trait	PC1	PC2	PC3
Blade Area	0.21	−0.20	−0.01
Blade Convex Hull Area	0.21	−0.17	0.02
Blade Convex Hull Perimeter	0.21	−0.19	0.00
Blade Length	0.20	−0.19	−0.05
Blade Perimeter	0.19	−0.17	0.08
Coverage	0.17	0.11	0.24
DAS 21 Height^a^	0.19	0.18	−0.12
DAS 21 Height Max^a^	0.18	0.18	−0.18
DAS 21 Leaf Area^a^	0.18	0.22	0.12
DAS 21 Leaf Area Index^a^	0.17	0.24	0.13
DAS 21 Leaf Area Projected^a^	0.18	0.23	0.12
DAS 21 Light Penetration Depth^a^	0.18	0.16	−0.15
DAS 28 Height^a^	0.18	0.26	−0.17
DAS 28 Leaf Area^a^	0.20	0.22	−0.02
DAS 28 Leaf Area Index^a^	0.18	0.24	−0.03
DAS 28 Leaf Area Projected^a^	0.19	0.23	−0.05
DAS 28 Light Penetration Depth^a^	0.17	0.29	−0.15
Leaf Number	0.09	0.09	0.83
Root Dry Weight	0.21	−0.01	0.09
Specific Leaf Area	0.20	−0.20	−0.02
Specific Leaf Convex Hull Area	0.20	−0.20	0.01
Specific Leaf Convex Hull Perimeter	0.20	−0.20	−0.01
Specific Leaf Length	0.20	−0.19	−0.04
Specific Leaf Perimeter	0.19	−0.15	0.13
Specific Leaf Width	0.20	−0.20	0.03
Specific Petiole Area	0.16	−0.15	−0.02
Stem Dry Weight	0.20	−0.07	−0.15
Stem Fresh Weight	0.19	−0.07	−0.17
Variance Explained (%)	72.40	11.40	3.30
Cumulative Variance (%)	72.40	83.80	87.10

^a^*DAS* Days after sowing

### Principal component reduction of yield traits

To further simplify the complexity of the 28 common yield associated traits, PCA was performed. The first three principal components (PCs) collectively explained 87.1% of the total variation in vegetative yield (Table 4, Supplementary Table 4), providing a reduced-dimensionality framework for interpreting trait contributions to yield.

PC1 (explaining 72.4% of the variance) captured the dominant axis of covariance among leaf blade size related traits (Tables 4 and 5). These traits primarily reflect biomass accumulation and organ growth of the leaf, corresponding to sub-module 1.2. Root dry weight also highly contributed to PC1. PC2 (explaining 11.4% of the variance) captured canopy coverage and plant density traits (Tables 4 and 5). This component emphasizes plant density and architectural traits that may influence light interception and space-use efficiency. These traits correspond to the earlier identified sub-module 2.2. PC3 (explaining 3.3% of the variance) reflect canopy structure associated with trait modules 4 and 2.1 (Table 5). These traits influence canopy organization of the leaves that may contribute to the overall plant productivity.

**Table 5:** Biological interpretation of principal components (PC1-PC3) based on the top two index traits, with each component mapped to its corresponding phenotype module

PC	Index traits	Biological importance	Trait module
1	Blade Area, Blade Convex Hull Area	Leaf blade size and expansion	Module 1.2
2	DAS 28 Light Penetration Depth, DAS 28 Height^a^	Plant density and vertical architecture	Module 2.2
3	Leaf Number, Coverage	Canopy structure	Module 4 & 2.1

^a^*DAS* Days after sowing

In total, the 113 quantitative traits could be functionally reduced to three important components. They each represent important trait areas that potentially contribute to the TFW of kale. The index traits of these three dimensions, in order of PC importance, are Blade Area, Light Penetration Depth at DAS 28 and the total number of leaves (Table 5).

## Discussion

### Phenotypic diversity and trait heritability under CEA

This comprehensive phenomics analysis of the global kale germplasm provides a detailed blueprint of its phenotypic landscape and generates a foundational phenomics resource. This study captured substantial phenotypic variation across 203 diverse kale accessions. The wide range of variation uncovered is consistent with previous studies that reported significant morphological and genetic diversity across different kale and other *B. oleracea* varieties [45–47]. High variation traits relating to leaf area, plant architecture, stem size and shape, canopy coverage, and root-to-shoot ratios, underscored broad morphological and architectural diversity among accessions. The moderate to high general h^2^ of many measured traits further indicates that a substantial proportion of this phenotypic variation is genetically determined under the CEA conditions tested in this study. Traits associated with organ size and biomass accumulation, including leaf area, stem weight, and root dry weight, exhibited the highest heritability, indicating that additive genetic effects explain a substantial proportion of their variation and suggesting strong potential for selection within breeding programs. In contrast, traits related to spectral and derivative indexes showed lower heritability, reflecting greater sensitivity to environmental and developmental influences. These findings suggest that while morphological traits offer strong targets for genetic improvement, specific physiological traits including hyperspectral indexes that describe plant hue affecting photosynthetic efficiency may benefit more from genotype-environment optimization that is largely possible within CEA systems.

The traits recorded in this study were measurable across the five different destructive and non-destructive phenotyping methods employed in this study. Such phenotyping methods are useful in quantifying the diverse phenotypic diversity that exists across *B. oleracea* species [48–50]. The observed diversity across traits highlights the strong breeding potential of the kale germplasm collection. While this experimental design utilized here supports the generation of a high-resolution phenomics resource, it necessarily limits the generality of conclusions across other CEA configurations or production regimes. The heritability measures here should be further validated in further multi-environment and multi-cycle experiments.

### Modular trait architecture and implications for CEA breeding

Seven biological trait modules, encompassing morphological, architectural, and hyperspectral traits across leaf, stem, root organs were identified by correlation clustering and illustrated the coordinated nature of plant traits. These modules reflect the phenotypic variability within the global kale germplasm collection. The trait modules defined in this study represent one of several possible organizational frameworks and are dependent on the chosen correlation threshold and clustering method. While the |r|≥ 0.6 threshold was selected to capture moderate to strong trait associations, alternative thresholds or clustering may yield differing module boundaries. Consequently, these networks should be interpreted as exploratory, hypothesis generating frameworks for understanding trait covariation rather than as definitive representations of causal relationships. Notably, negative correlations between sub-modules 1.1 (organ shape) and 1.2 (organ size) suggest an inverse association between plant organ size and shape. For example, accessions with larger leaves tend to display less extreme leaf margin variation. Conversely, positive correlations between expansive canopy growth (Module 2.2) and compact architecture (Module 2.1) suggest that these traits co-occur across accessions. While our dataset does not provide mechanistic evidence of causal trade-offs, these correlational patterns are nonetheless relevant for breeding decisions. In the context of CEA, where planting density, canopy layering, and light distribution are tightly managed, trait combinations may map to different variety ideotypes. For example, compact leaves with high solidity may contribute to denser canopies that maximize space use but risk shading lower leaves if leaf area is not simultaneously optimized. Large but more dissected leaves may also improve internal canopy light penetration but reduce packing efficiency at high density. Such relationships imply that breeders may need to prioritize trait modules depending on production goals and harvesting practices. Negative correlations may constrain simultaneous trait improvement. As such, index-based or multi trait selection may be more effective than independent trait selection. Alternatively, breeders may target module-level genetic variation to shift trait combinations in a coordinated manner.

In other staple crops (soybean, sugarcane and wheat [51–53]), the variation in canopy density and architecture have been shown to affect light capture efficiency and plant yield. While their functional relevance in leafy vegetables and CEA environments remains to be tested, our findings contribute to evidence that similar patterns of physiological behavior may be available and can be applied in leafy vegetables. The application of relationships uncovered in outdoor field environments should be validated and applied within the context of CEA. Module 6.1 (spectral indices) and 6.2 (seedling architecture) illustrate temporal and developmental distinctions in physiological and structural traits captured by high-throughput imaging. The differences between patterns in seedling architectural traits at 14 DAS (Module 6.2) and those at 21 and 28 DAS (Module 2.2), suggest distinct early versus later vegetative growth strategies. Comparable early to late growth pattern differences have also been reported in *Arabidopsis* [54] and rice [55]. Further specific physiological and mechanistic testing should be performed to confirm the biological basis of these patterns of correlation.

Modules 3 (Leaf Shape), 4 (Leaf Number), 5 (Leaf Margin) and 7 (Leaf Orientation) all represent clusters of leaf specific traits. Collectively, they explain the physiology and shape of the leaves. Canopy coverage and density were closely associated with leaf orientation and number, demonstrating that these traits vary together across accessions. Similar modular trait organization has been reported in sorghum and wheat phenomics studies, where architectural and canopy traits were primary determinants of biomass accumulation [20, 56]. Within CEA farm systems, architectural traits such as leaf angle and canopy coverage are recognized as critical for improving yield, particularly under high-density planting conditions typical of vertical farms [16, 17]. The modular framework and foundational resource presented here provides a basis for prioritizing suites of complementary traits rather than focusing on individual traits in isolation. These modules will be valuable for future genetic analyses to characterize the genomic architecture underlying phenotypic variation and further breeding programs [45, 49, 57–59].

### Candidate yield targets for CEA breeding

In CEA farm practices in Singapore, entire kale plants are often grown to about 50 DAS before they are harvested in their entirety. The dense, indoor CEA nature of farms prevent farmers from outgrowing kale for long periods of time and practicing longer cycles or multi-cut harvest regimes. Recent studies have captured kale phenotypes in an indoor cultivated environment up to 45 and 34 days respectively [60, 61]. Furthermore, given the limitations of the Planteye Phenotyping system, TFW at DAS28 was used as a representative measure of vegetable yield since the above ground mass of kale constitutes an edible portion [9]. We note that such data hence largely applies to baby leaf and juvenile plant harvest systems, and the conclusions made here may not directly relate to longer regime harvest cycles or multi-cut harvest systems. Integrative analyses combining PLS-VIP and differential trait analysis identified key phenotypes associated with vegetative yield in leafy vegetables. Key identified phenotypes include leaf size, stem weight, plant architecture, and root size related traits. Principal component analysis distilled these 28 traits into three orthogonal components. These components, corresponding to trait modules 1.1, 2.2, and a composite of modules 2.1 and 4 summarize yield variation along trait axes. Previous studies have shown that leaf size is associated with overall plant biomass allocation [62] and canopy structure influences light use efficiency in other species [63]. Notably, leaf size has been shown to be strongly genetically driven [64]. Interestingly, Leaf Number exhibited a high PLS-VIP score yet contributed a comparatively low loading on PC1. While PLS-VIP captures traits most predictive of yield, PCA summarizes dominant sources of phenotypic variation. As a result, Leaf Number could play a pivotal role in predicting biomass accumulation, even though its absolute variance among accessions is limited relative to organ size traits. While Leaf Number may accurately predict TFW, it’s limited phenotypic variance indicates there may be limited capacity for breeding improvement as compared to leaf size. Consequently, breeding strategies that target organ level traits may achieve greater improvements in yield potential. In *Arabidopsis* and *Brassica rapa*, root and shoot architecture phenotypes have also been shown to jointly influence growth outcomes, reflecting the complex repertoire of traits contributing to biomass allocation [56, 65]. Importantly, canopy light penetration traits were also identified as important for yield in CEA systems for lettuce and spinach [7, 8]. They highlight the critical role of canopy structure in optimizing light-use efficiency under artificial illumination. Despite these findings, functional significance cannot be inferred from morphology alone, and direct physiological measurements remain necessary to verify these relationships.

Comparable PCA-based approaches in rice and wheat have similarly emphasized that a small number of latent trait dimensions explaining much of the available variation in yield performance [20, 66]. Similar phenomics-based machine learning based approaches have been used to predict biomass and leaf area in wheat [67, 68]. The increasing integration of machine learning information technology with phenomics data will further streamline the complexity and duration of trait selection in kale and related leafy vegetables to improve breeding cycles.

The traits highlighted here underscore selection targets relevant for CEA systems, where maximizing harvestable yield is essential. Beyond increasing leaf size, combining traits that contribute to compact plant architecture and efficient canopy organization may enhance performance in high-density CEA configurations. These findings align with emerging recommendations for breeding leafy vegetables specifically for CEA constraints [11, 16, 17]. The moderate to high h^2^ estimates reinforce the potential for genetic improvement under CEA. Overall, this study provides a phenomics foundation for trait prioritization in kale, serving as a resource for future breeding and genetic analyses.

### Limitations and future directions

Despite the breadth of phenotypic data captured, several limitations should be considered. First, the analyses were conducted within a single CEA system. While CEA reduces environmental variation, the absence of multi-environment, multi-cycle, or multi-density assessments limits our ability to evaluate the stability of trait relationships and module structures across different environments. Consequently, h^2^ estimates reflect only the additive genetic variance captured by the genomic relationship matrix. Multi-environment and multi-cycle trials will therefore be essential to fully partition genetic, environmental, and genotype-by-environment (G × E) components. Future work incorporating varied environmental and harvesting will be necessary to test the robustness of observed phenomics patterns. Second, while this study establishes a comprehensive phenotypic baseline, it does not resolve the mechanistic genetic or physiological pathways underlying the observed trait patterns. The genomic data generated here provide a basis for downstream GWAS, genomic prediction, and population analyses. Further integration with transcriptomics, metabolomics, and targeted physiological assays could make available even deeper biological findings and hypothesis testing. Finally, the study did not include direct measurements of physiological performance which are necessary to determine whether morphological or spectral associations correspond to functional differences. Although imputation enabled integrated multivariate analyses, formal sensitivity testing was not performed, and results should therefore be interpreted as descriptive patterns. These limitations highlight important directions for future research and reinforce the value of this dataset as a hypothesis generating resource for advancing kale and leafy vegetable breeding in CEA.

## Conclusions

In conclusion, this study establishes a comprehensive phenomics framework for kale, dissecting its trait architecture and identifying the key drivers of vegetative yield in CEA. By identifying consensus yield associated traits and reducing them into interpretable components, we provide a pathway for phenomics informed breeding of leafy vegetables. Integration of these phenotypic insights with genomic resources [19] will accelerate the development of cultivars optimized for the unique demands of CEA. Based on our integration of machine learning with phenomics, we recommend the improvements of traits related to kale leaf area, leaf density and canopy structure to improve yield for CEA production. These findings establish a comprehensive phenotypic framework and provide a foundational phenomics resource for kale.

## Supplementary Information

Supplementary Material 1.

## Data Availability

The datasets used and/or analyzed during the current study are available from the corresponding author on reasonable request. All the analysis tools used during this study have been included in the Materials and Methods.

## Abbreviations

BA: Blade Area
BCHA: Blade Convex Hull Area
BEI: Blade Ellipticalness Index
BSOL: Blade Solidity
CEA: Controlled Environment Agriculture
CV: Coefficient of variance
DAS: Days After Sowing
DBM: Digital Biomass
FC: Fold Change
FDR: False Discovery Rate
GWAS: Genome-Wide Association Study
G × E: Genotype by Environment Interactions
HTP: High-Throughput Phenotyping
h^2^: Narrow-sense heritability
LAN: Leaf Angle
LA: Leaf Area
LAI: Leaf Area Index
LAP: Leaf Area Projected
LI: Leaf Inclination
LINT: Leafing Intensity
LPD: Light Penetration Depth
NDVI: Normalized Difference Vegetation Index
PCA: Principal Component Analysis
PLS-VIP: Partial Least Squares Variable Importance in Projection
PSRI: Plant Senescence Reflectance Index
r: Pearson Correlation
RA: Root Area
RDW: Root Dry Weight
RLLA: Root Length-Leaf Area Ratio
RPAR: Root Perimeter-Area Ratio
RST: Root-Stem Ratio
RWL: Root Width-Length Ratio
r^2^: Coefficient of Determination
SD: Standard Deviation
SDW: Stem Dry Weight
SF: Surface (pixels)
SFW: Stem Fresh Weight
SLA: Specific Leaf Area
SLCHA: Specific Leaf Convex Hull Area
SNP: Single Nucleotide Polymorphism
SRA: Specific Root Area
SRL: Specific Root Length
STA: Stem Area
STCHA: Stem Convex Hull Area
TFW: Total Fresh Weight
