# Supplementary material for: Comprehensive phenomics and vegetative yield analysis of global kale (Brassica oleracea var. acephala) germplasm in controlled environment agriculture
Source: BMC Plant Biol. 2026 Feb 17;26:539. doi: 10.1186/s12870-026-08380-6 (PMC13014718; doi:10.1186/s12870-026-08380-6)
Supplement: Supplementary file 1 — Supplementary Material 1. [file 12870_2026_8380_MOESM1_ESM.zip › Supplementary Figure 1.docx]

Supplementary Figure 1. Trait correlation heatmaps for Modules 1-9. (a) Module 1. (b) Module 2. (c) Module 3. (d) Module 4. (e) Module 5. (f) Module 6. (g) Module 7. (h) Module 8. (i) Module 9.

(a)

(b)

(c)

(d)

(e)

(f)

(g)

(h)

(i)
